# The Pilgrimage on the Camino de Santiago and Its Impacts on Marital and Familial Relationships: An Exploratory Study

**DOI:** 10.1007/s10943-023-01825-4

**Published:** 2023-05-01

**Authors:** Sławomir Tykarski, Franciszek Mróz

**Affiliations:** 1 Faculty of Theology, Nicolaus Copernicus University in Toruń, ul. Gagarina 37, 87-100 Toruń, Poland; 2 Department of Tourism and Regional Studies, Institute of Geography, Pedagogical University of Krakow, ul. Podchorążych 2, 30-084 Kraków, Poland

**Keywords:** Camino de Santiago, Faith, Family, Family ties, Health, Marriage, Pilgrims, Pilgrimages

## Abstract

This paper presents the results of a survey conducted among people walking the pilgrimage route to the shrine of St James in Santiago de Compostela. The aim of the research was to investigate how a pilgrimage on the Camino de Santiago among a married couple or family affects marital or familial relationships, whether walking the trail to Compostela together influences the behaviour of family members towards each other and whether it forms positive attitudes and behaviour. The empirical basis of the study is the results of a survey among respondents who made the Camino de Santiago pilgrimage, as well as in-depth interviews conducted with 24 spouses of pilgrims along the Way of St James. The study revealed that doing the pilgrimage as a married couple or family had a positive impact on intra-family relationships. According to the interviewees, going on the pilgrimage together helped to strengthen marital bonds and trust, improve communication and mutual connection, show care and affection and improve contact with children.

## Introduction

One of the most characteristic trends taking place in the European pilgrimage space in the last two decades is the renaissance of hiking and pilgrimage along the route to the Cathedral of St James in Santiago de Compostela (Amaro et al., 2018; Herbers & Plötz, 1996; Margry, 2015; Mróz, 2021; Onorato & Rizzi, 2017; Roszak, 2019). The relics of St James the Elder—a disciple of Jesus Christ and the first martyr in the group of Apostles—are kept in this temple, according to Christian tradition and the recent research of many scholars (Alarcón, 2017; Baliñas, 2017; Barral, 2016; Roszak, 2022a). It is estimated that in the thirteenth century, 500,000 pilgrims made the pilgrimage to the shrine of St James in Santiago de Compostela each year (Mróz, 2020; Murray & Graham, 1997). The renaissance of the pilgrimage route to Compostela began in the early 1980s. In 1987, the Council of Europe recognised the Camino de Santiago as the first European Cultural Route, and the route was added to UNESCO’s list of World Cultural and Natural Heritage in 1993 and 2015 (Spanish territory) and in 1998 (French territory) (Mróz, 2019; Mróz et al., 2019; Roszak, 2017).

**Fig. 1:**
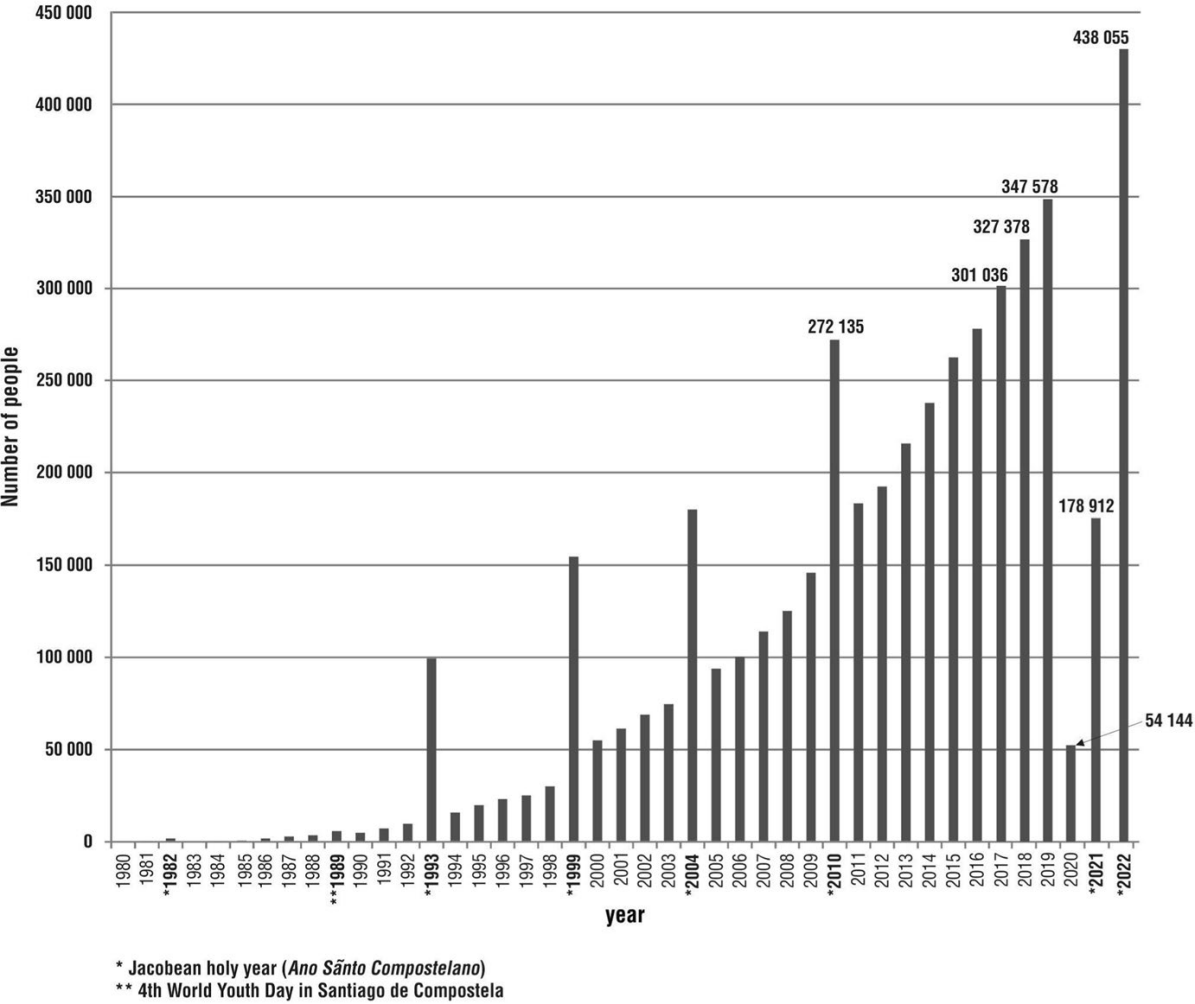
Pilgrim/tourist traffic to Santiago de Compostela between 1980 and 2022, as recorded at the Santiago de Compostela Pilgrimage Office Based on data from the Santiago de Compostela Pilgrimage Office (*Oficina de Acogida al Peregrino*) www.oficinadelperegrino.com/estadisticas

The Way of St James is recognised as the most innovative, expansive, inspiring and consolidated pilgrimage and cultural route in the world. The development, success and worldwide prominence of the Camino de Santiago has had a significant impact on the restoration and development of other ancient (not only Christian) pilgrimage routes: Via* Francigena*, the Jerusalem Way, the Way of St Olaf, the Way of St Siegfried in Sweden, the Way of Sts Cyril and Methodius, *Romea Strata*, *the Pilgrims’ Way* from Winchester to Canterbury and, in Japan, *Shikoku Henro* and *Kumano Kodō* (Corcho Sánchez & Gómez-Ullate, 2019; Duda, 2016; Jørgensen et al., 2020; Kato & Progano, 2017; Krogmann et al., 2021; Maak, 2010; Margry, 2015; Øian, 2019; Progano & Kato, 2018; Seryczyńska, 2019; Vistad et al., 2020). Today, the Camino de Santiago network in Europe is more than 80,000 kms of marked route (Mróz, 2020). In 2022, a record number of people for recent history walked this route in the Iberian Peninsula alone. Indeed, in 2022, the Pilgrimage Office in Santiago de Compostela (Oficina de Acogida al Peregrino, 2023) registered 438,055 people, a quarter million more than in 2021 and almost 100,000 more than in 2019 (before the outbreak of the COVID-19 pandemic) (Mróz, 2021) (Fig. 1). No other pilgrimage and cultural route in the world can boast such a spatial reach as the Camino de Santiago.

The cultural, religious, social, economic and geographical phenomena taking place in the pilgrimage space of the Camino de Santiago are of scholarly interest to researchers from fields such as history, geography, theology, sociology, ethnography and cultural studies (Amaro et al., 2018; Doburzyński, 2021; Duda, 2015; Foster, 2018; Gusmán et al., 2017; Kotecki, 2017; Krogmann et al., 2013, 2016; Marszałek, 2017; Mieck, 1978; Mróz, 2017, 2019; Mróz et al., 2019; Roszak, 2017, 2019, 2022b; Rucquoi, 2018; Tanco, 2011; Wyrwa, 2012). The pilgrimage space of the Camino de Santiago is dynamic and diverse. In an ‘interior’/spiritual sense, it is a space of encounter with God, the saints and the blessed (St James) and above all, as the caminos emphasise, a space of encounter with oneself—a journey into the ‘depths of oneself’. The Camino de Santiago, as those returning from Santiago de Compostela say, is a route on which ‘the old man dies and the new man is born’, a route of conversion and humility, a ‘way of life’. The Way of St James is a route for individual reflection on one’s life so far and a search for potential transformation (Blom et al., 2016; Nilsson, 2016).

In an external sense, the pilgrimage space of the Camino de Santiago is a space of encounter with others, those with whom the pilgrim walks the route, including but not limited to spouses, children, friends and—absolutely unsurprising on the Camino—animals such as horses, donkeys or dogs. (One example is the extremely eloquent and unique photograph by Claudio Modola of an Italian pilgrim kneeling and thanking his horse, Cortez, for 44 days of walking and riding the Camino de Santiago to the tomb of St James, without whom he would not have been able to complete the journey.) The Camino de Santiago is also a space of encounter with the pilgrims encountered on the route. Finally, the pilgrimage space of the Way of St James is one in which to encounter the geographical environment—especially nature and culture, tangible and intangible heritage and the local community, the people living and working along the route (Kaszowski, 1996).

Pilgrimage—both individual and collective—is a highly sensory experience, because both on the way to the holy place—in the pilgrimage space—and within the sanctuary space, pilgrims process visual, auditory and tactile stimuli, observing and participating in a variety of rituals and acts of worship (Faria et al., 2022; Sigala, 2020). Agapito et al. (2013) believe that a multidisciplinary approach to the human senses (sight, hearing, smell, taste and touch), demonstrates their importance for individual experience and perception of space. Sensory stimuli influence behaviour, and ‘being on the road’ promotes multisensory encounters (Agapito et al., 2013). Experiences on the Camino de Santiago affect all human spheres: intellectual, physical, emotional and spiritual. Individual as well as group pilgrimage is a significant (unique) experience for each pilgrim, which remains in the memory for a long time. In fact, a pilgrimage is a unique time to reconnect with one’s values, to talk to God, to get to know the world and oneself, to gain strength, to renew oneself mentally and spiritually (experience *catharsis*), to be with others on the way and to realise one’s freedom.

One of the distinctive features of modern long-distance trail pilgrimage, a category to which the Camino de Santiago belongs, is that pilgrims value the experience of walking—of making the journey—more than that of eventually reaching a holy place (Amaro et al., 2018; Devereux & Carnegie, 2006; Progano & Kato, 2018; Vistad et al., 2020). Caminers very often emphasise that ‘it is not the destination that is most important, but being on the way, being on the move, and taking one more step’ (Mróz & Matuszczak, 2019).

It is worth noting that the idea of pilgrimage is related to the term *homo viator* (‘pilgrim man’), known even in ancient literature or philosophical currents. The term has also taken root in Catholic theology. It can be found in the pages of the Holy Scriptures. The biblical vision of pilgrim man oscillates around the theme of transition from earthly to eternal life, and concerns pilgrimage understood as a process of striving for perfection or personal holiness (Dec, 1985). Pilgrimage as a family on the Camino de Santiago corresponds with this understanding of *homo viator*. The pilgrim family, as discussed in the teachings of Vatican II, reflects the people of God wandering through earthly life towards an encounter with God in eternity (Anthony, 2018; Roszak, 2011, 2014; Second Vatican Council, 1964). In addition, overcoming the hardships of pilgrimage, struggling with one’s weaknesses and helping one’s spouse or children can contribute to the formation of the pilgrim’s character and spiritual growth, the experience of ‘being for the other’ and discovering the fullness of humanity in the formation of an attitude of love, sensitivity, compassion or selflessness (Łużyński, 2021).

The study aims to investigate how marital/familial pilgrimage on the Camino de Santiago affects marital/familial relationships, whether walking the trail to Compostela together influences the behaviour of family members towards each other and whether it forms positive attitudes and behaviour (Tykarski, 2019). Surprisingly, with the exception of the work of Kathleen Jenkins (2021), there has been no research addressing this issue in the extensive literature on the Camino de Santiago. The work presented here fills this research gap.

## Materials and Methods

The main empirical basis of the study is a survey among respondents who made the Camino de Santiago pilgrimage (Snow, 2022). The study used a qualitative method of diagnostic survey, and the tool was an interview questionnaire consisting of 32 questions (closed questions with both quantitative and categorical answers—disjunctive and conjunctive criteria). The respondents were selected by means of a network selection method of surveying only those people who had made a pilgrimage along any section (domestic or international) of the Camino de Santiago. It is therefore a non-random sample, so the research is not representative. The surveys were anonymous and collected through an online questionnaire. Respondents were given a link which they could follow to complete the questionnaire. The period of the research was 28 days, from 6 October to 2 November 2022. After this time, the link to the questionnaire was deactivated. Using the aforementioned tool, it was possible to recruit 30 respondents. It should be noted that not everyone answered the entire set of questions.

Determining how marital or familial pilgrimage on the Camino de Santiago affects marital or familial relationships was also possible thanks to in-depth interviews conducted with 24 people—spouses of pilgrims on the Way of St James—and thanks to the personal experience of one of the authors. In-depth interviews are considered one of the most important and widely used research tools in qualitative research (Berezowski, 1980; Berg, 2001; Goodman, 2001; Tutty et al., 1996). They provide a reliable pathway to understanding the processes and factors that determine the development, stagnation or regression of a given spatial phenomenon (Goodman, 2001). We have been conducting field research—observations and in-depth interviews—in the Camino de Santiago pilgrimage space since 2010. This is because we have been organising and participating in group or family pilgrimages along the Way of St James since that time. The trust of the pilgrims gained during this time resulted in their willingness to express their experiences and answer our questions honestly. Of course, we pledged to maintain confidentiality, and we also focussed a great deal of attention on ensuring that the interviews were not distorted in any way by our perspective. For the purpose of the stated goals of the research, we conducted structured in-depth interviews while on pilgrimage along the Spanish, French, Lithuanian and Polish sections of the Way of St James between March and September 2022. The interviews, with the consent of each interviewee, were recorded on a dictaphone and transcribed literally.

The study was performed in line with the principles of the Declaration of Helsinki, and according to local legislation and national guidelines on research involving human subjects. All quotes resulting from this research were provided to the researchers with the explicit consent of the participants. The research we conducted was approved by the Faculty Research Ethics Board of the Faculty of Theology of Nicolaus Copernicus University in Toruń (certificate number 1/2023).

The material collected during the study was organised and statistically analysed in graphical and tabular form. Graphical presentation methods were used; descriptive analytical and comparative methods were mainly used to present the results.

### Profile of Respondents

Thirty people participated in the study, including 22 women (73%) and eight men (27%). All respondents to the survey were from Poland.

Among the study population, 20 people (67%) had a university degree and 10 people (33%) had a secondary education. There were no participants with vocational or primary education (Table 1).

**Table 1:** Demographic characteristics of the participants. *Source* The authors, on the basis of the survey conducted

	Women				Male					
Gender	22				8					
Nationality	Polish nationality (100%)				Polish nationality (100%)					
Education status	22				8					
	0	0	8	14	0	0	2	6		
	Primary education	Vocational education	Secondary education	Higher education	Primary education	Vocational education	Secondary education	Higher education		
Religious denomination	Catholicism (100%)				Catholicism (100%)					
Relationship to faith	22				8					
	8	14	0	0	0	5	2	1	0	0
	Deep believer	Believer	Undecided	Indifferent	Disbeliever	Deep believer	Believer	Undecided	Indifferent	Disbeliever
Assessment of the material situation	22				8					
	2	14	6	0	0	1	3	4	0	0
	Very good	Rather good	Average	Rather bad	Very bad	Very good	Rather good	Average	Rather bad	Very bad

When asked what type of relationship the respondents were in, 29 people (97%) ticked the option ‘sacramental marriage’ (so-called ‘church wedding’) and one (3%) chose ‘non-religious marriage (i.e. contracted outside the Catholic Church). None of the respondents were in a civil union or single.

The fertility issue of the surveyed marriages is as follows. The largest group (12 respondents; 40%) indicated that they had two children. This was followed by eight respondents (27%) with three children, four respondents each (13%) with four children and a single child and one person (3%) with five children. One participant did not answer this question.

Regarding religious denomination, 100% of the respondents were of Catholic faith, of which 16 people (53%) described their attitude towards the faith as believing, 13 (43%) as deeply believing and one person who was undecided but attached to a religious tradition.

The respondents had the following professions: the largest number of respondents were pensioners (six; 20%), followed by teachers and office workers (four each; 13%). The respondents also included an IT specialist, an educator, a warehouse logistics manager, a speech therapist, an architect, a dressmaker, a chemical laboratory specialist, a midwife, a farmer, a therapist, a guide and a massage therapist/physiotherapist. One person did not work professionally and three respondents (10%) did not indicate a profession.

Regarding the assessment of their financial situation, the most frequent choice of the respondents was ‘rather good’ (17; 57%), followed by ‘average’ (10; 33%) and ‘very good’ (10; 10%). There were no respondents who were in a bad or very bad material situation.

## Results

When asked how the pilgrims made the journey, the largest group (47%) indicated with their spouse, 30% separately, 20% journeyed part of the way with their spouse and part separately and one person marked the option ‘other’.

The respondents most often (53%) made the pilgrimage without their child(ren), 30% with their child(ren), 10% part of the way with their offspring and part without and 7% of respondents selected the option ‘other’.

When asked how they mostly travelled on the Camino de Santiago, 97% said on foot and only one person (3%) was travelling by bicycle.

**Fig. 2:**
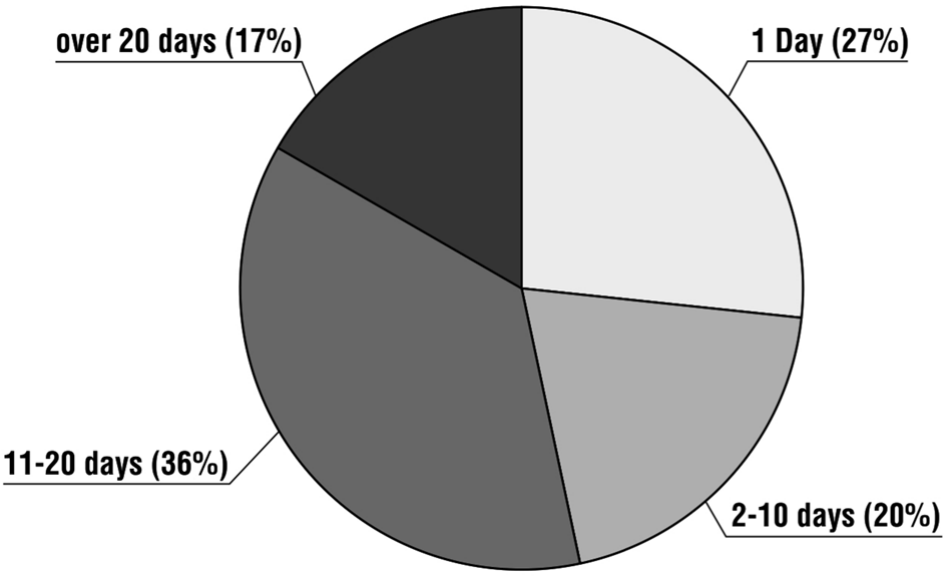
Distribution of respondents’ answers to the question, ‘On average, how long was your Camino de Santiago pilgrimage?’

The distribution of average pilgrimage time varied considerably. For eight respondents (27%), these were one-day pilgrimages. For six respondents (20%), the duration of the pilgrimage was between 2 and 10 days, 11 pilgrims (35%) spent between 11 and 20 days on the route, while five people (17%) spent more than 20 days (including about a month or more). There were cases of pilgrims hiking for weeks at a time (Fig. 2).

**Fig. 3:**
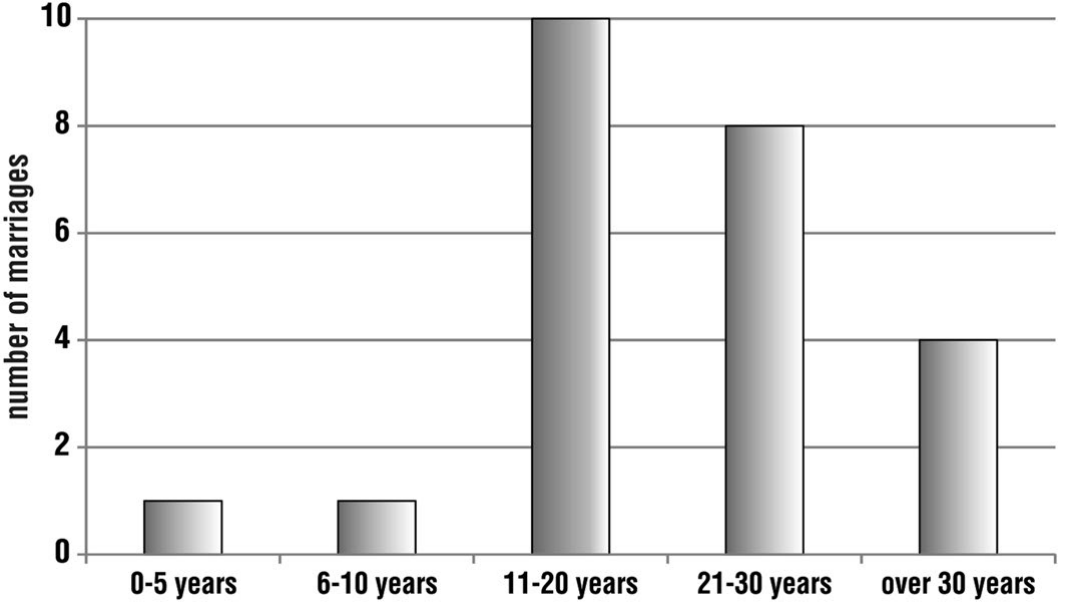
Distribution of respondents’ answers to the question, ‘Please indicate how long you have been married’

Twenty-four responses were received about the duration of marriage of pilgrims walking the Camino de Santiago. The largest group is made up of those with marital seniority between 11 and 20 years (10 people). This is followed by 21–30 years (8 people), over 30 years (4 people), up to 5 years (one person) and 6–10 years (one person). It can be seen that the most frequent pilgrims were those with medium and long marriages. Quite a few of the respondents going on the pilgrimage had been married for more than 20 years. There were also four respondents who had more than 40 years of marriage (Fig. 3).

The interview questionnaire also included a question about the age of the children whom the respondents took on the pilgrimage. The most common age range was between 13 and 18 years (12 responses). This was followed by children aged 6–12 years (8) and 0–5 years (5). There was also no shortage of respondents admitting that adult children went on the pilgrimage with them (4 responses).

### Motives and Intentions of Pilgrimage

**Fig. 4:**
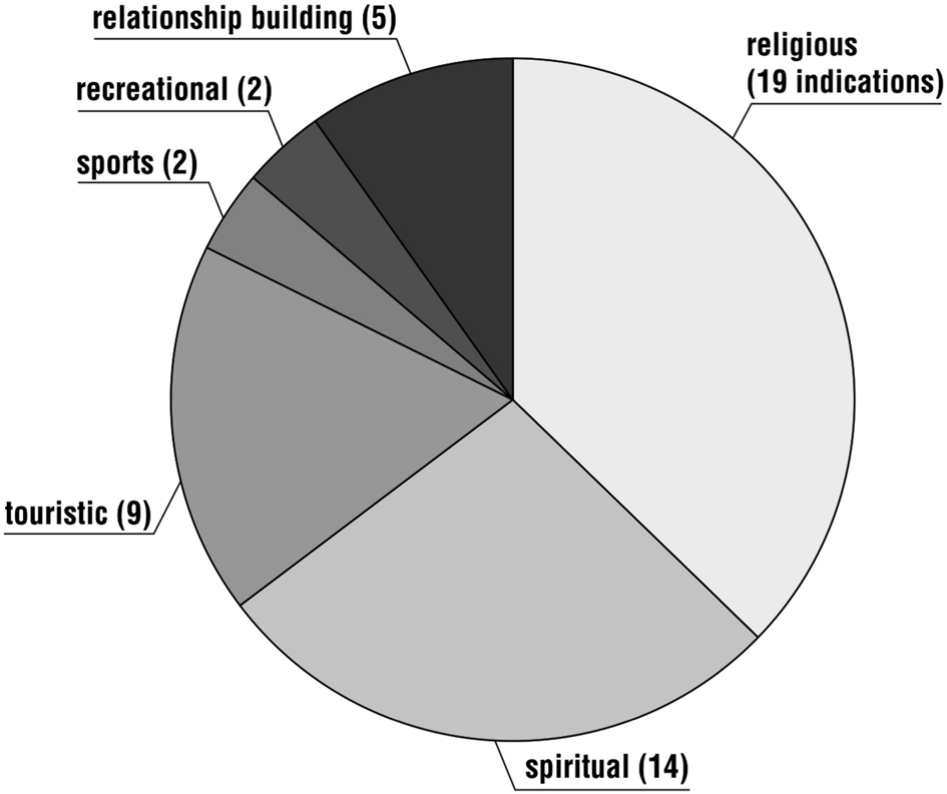
Distribution of respondents’ answers to the question, ‘Please write what motive (e.g. spiritual, religious, touristic, recreational or sports) guided your decision to make the pilgrimage’

The respondents were asked about the motive(s) behind their decision to go on a pilgrimage; the question was multiple-choice. The most common reason for pilgrimage was religious (19 responses), followed by spiritual (14), touristic (9) and recreational or sporting (2 each). There were also responses (5) that the motive for the pilgrimage was to strengthen family ties, to build relationships with spouses or children and to socialise with other pilgrims (especially after the period of global isolation caused by the COVID-19 pandemic) (Fig. 4).

The questionnaire asked about the intention behind the pilgrimage. The most frequent one was for the family, blessings for the family, the spouse and children, healing of relationships and mutual reconciliation (21 responses). The second, in terms of frequency, was an intention for health (eight). This was followed by giving thanks, world peace, intentions carried over from other trusted people unable to make the journey themselves (four responses each), for strengthening existing faith (three), for the deceased and souls in purgatory (two), for help in making the right choices (two) and in order to witness faith through pilgrimage (two). There were also single answers about intentions such as healing from addictions, conversion or asking for blessings in daily life. It is noteworthy that some pilgrims had a main intention to which they dedicated the whole pilgrimage, while others changed their intention during a particular part of the journey. In an in-depth interview, one respondent said, ‘I chose St Paul’s “Hymn of Love” as the motto for my pilgrimage on the Camino de Santiago. I wanted to dedicate this time to reflecting on my love. Love in general, but in particular my love for my husband’ (P1). However, there is no doubt that the family intention was the dominant one. This underlines how important it is in the lives of pilgrims.

### Forms of Marital Support During the Pilgrimage

The spouses gave each other support during their journey on the Camino de Santiago. Two varieties of support can be distinguished: physical and psychospiritual. The former consisted of couples simply being present for one another, walking together, making food, preparing equipment, massaging legs, applying bandages and planning the journey together. As far as the latter type of support is concerned, it is possible to have conversations and take the initiative of dialogue, motivate one’s spouse to continue the journey when weakened, show love and care (e.g. for the other’s health or fitness) and pray individually and together. In situations where only one spouse was on the pilgrimage, the other spouse’s help consisted of caring for the children or for ailing, elderly parents. It should be stated that, on the basis of the respondents’ answers, the most common form of mutual help between spouses during the pilgrimage was conversation and mutual prayer.

The respondents reported a lot of conversation during the Camino pilgrimage. The most common topics related to marriage and family were the children, their behaviour among their peers, how to raise them and their future. When a family was making the pilgrimage together, they had the opportunity to learn about a hitherto undiscovered side. One statement from the survey is worth quoting: ‘Mainly, there were topics related to our son, whom we were able to get to know from yet another side—his delight in every little thing’ (P2).

The conversations also revolved around the marital relationship, especially regarding issues of love, fidelity, forgiveness, respect, consent, support, needs, health, shared leisure time, personal or shared passions, marital problems, life choices, memories and life plans, the state of the world and current worries. As one interviewee recalled: ‘The road allowed us to distance ourselves from the sometimes tense and confusing daily life and to talk to other spouses …, which in turn allowed us to draw conclusions about our mutual marital behaviour, what causes pain or annoyance in our relationship and what we can do about it. Being on the road with other couples allowed for a deeper realisation that marriage does not have to be perfect and flawless in every dimension, that storms and downfalls happen everywhere and that this is a nearly universal experience which is meant to be worked through’ (P3). Some interviewees said that their conversations were about basically any topic, and that they returned to some of them more than once. Others did not talk about marriage and family or otherwise talked little with each other, focussing instead on prayer.

When asked in what ways the conversations and mutual activities during the pilgrimage differed from everyday ones, some people indicated that they did not notice any difference. One respondent wrote that the conversations were ‘not really different; the pilgrimage is a continuation of our lives. Just a kind of “retreat on the road”’ (P4). Others, however, noted differences. One respondent stated that ‘on pilgrimage we finally had time to talk to each other’ (P5), while another wrote that ‘during the pilgrimage we have time just for ourselves, far away from our daily responsibilities, so we can focus on talking’ (P6). In addition, the respondents emphasised the value of the pilgrimage in allowing them, through being surrounded by the beauty of nature and contact with God, ‘to forget, at least for a while, the worries of everyday life’, their duties and ‘everyday problems’.

The respondents also found that the Camino allowed them to spend more time with their spouse and to talk frankly about important things (feelings or needs) and about ‘topics rarely discussed before’. Conversations were conducted more calmly and lasted longer. At times, the road offered ‘a tranquility and peace, which brightened and calmed thoughts and emotions’. In an in-depth interview, one respondent stressed that the pilgrimage on their 25th wedding anniversary was particularly important and immensely enriching for her marriage: ‘We consider this pilgrimage as a thanksgiving for our marriage. For those few days we were alone with each other. It was a time to reflect on our marriage, on ourselves, on our family’ (P7).

It is worth quoting one pilgrim’s extensive statement: ‘We were, as it were, condemned to each other and to talk, which in a situation of marital difficulties is a challenge from which there is no possibility of escape as there is at home. The conversations were balanced, thoughtful and, more than on a daily basis, free of extreme negative emotions. On top of this, the long conversation, the lack of rush and the composure were big plusses. In addition, the pilgrimage in nature had a positive effect on us, released positive feelings, calmed us down, gave us the joy of being together in a beautiful place, served the purpose of conversation, while at the same time the road allowed for partial seclusion and did not force us to be together at all times. Talking during the hike was a chance to show sincerity, but it also triggered a greater need to look for everything that unites us and not just point out what divides us’ (P8). In addition, some respondents noted that the pilgrimage allowed them to connect with other married couples and other pilgrims, which allowed them to see their own marriage in a new light.

### Pilgrims’ Personal Reflections on Marriage and Family

It is interesting to analyse the personal thoughts the respondents had about marriage and family during the pilgrimage (or after the pilgrimage). First of all, they expressed their joy and gratitude for the opportunity to spend time together, the conversations held, getting to know and understand each other better and seeing that God, faith and family are the most important values in life. They also perceived the need to spend more time together. This was evidenced by the following statements: ‘I am happy to spend most of my time with my loved ones. It is a great gift that I appreciate’ (P4); ‘I was able to be with my wife again. I have something to thank God for again’ (P8); ‘I thanked God all the time for my husband, children and grandchildren’ (P9); ‘I am lucky to have such a family’ (P10); ‘I want to go to the end of the world with my wife’ (P11); ‘I thought about how important each member of our family is and each of us was called to something different’ (P12); ‘In order for the family to be strong and for us to be close to each other, we need to take care of each other and create opportunities to spend good time together. The daily problems that weaken the love in the family seem smaller when we all turn to God together, who gives us strength and power indeed’ (P13). One respondent noted that ‘it would be worthwhile to make a pilgrimage together and share everything, but sometimes this is not possible. Everyone has different talents and interests and wants to develop them. You can only talk about everything when you get back from the road. This is also how a community can be created’ (P14).

The respondents also noted that the pilgrimage allowed them to get to know their children better and fuse their relationships with them. This is illustrated by the following statement: ‘For me, above all, it was a great opportunity to get to know my son. I was delighted with how brave, courageous and interested in the world and people he is. It has been hard at times, but it has also had its value to get to know him in such difficult situations’ (P2). There was also a response in which a person noted the need to change their behaviour in their relationship with their children, to accept their individuality and their right to their time. Someone else, meanwhile, had thoughts on marital integrity in terms of raising children.

Sometimes the answers did not relate to the topic of marriage and family, but the thoughts were on the topic of God, faith, the joy of pilgrimage, love, respect, understanding, life goals, improving one’s life and planning the next pilgrimage. There was also one statement in which the person did not notice that the pilgrimage had changed anything in their understanding of marriage.

### Prayer Life During Pilgrimage

**Fig. 5:**
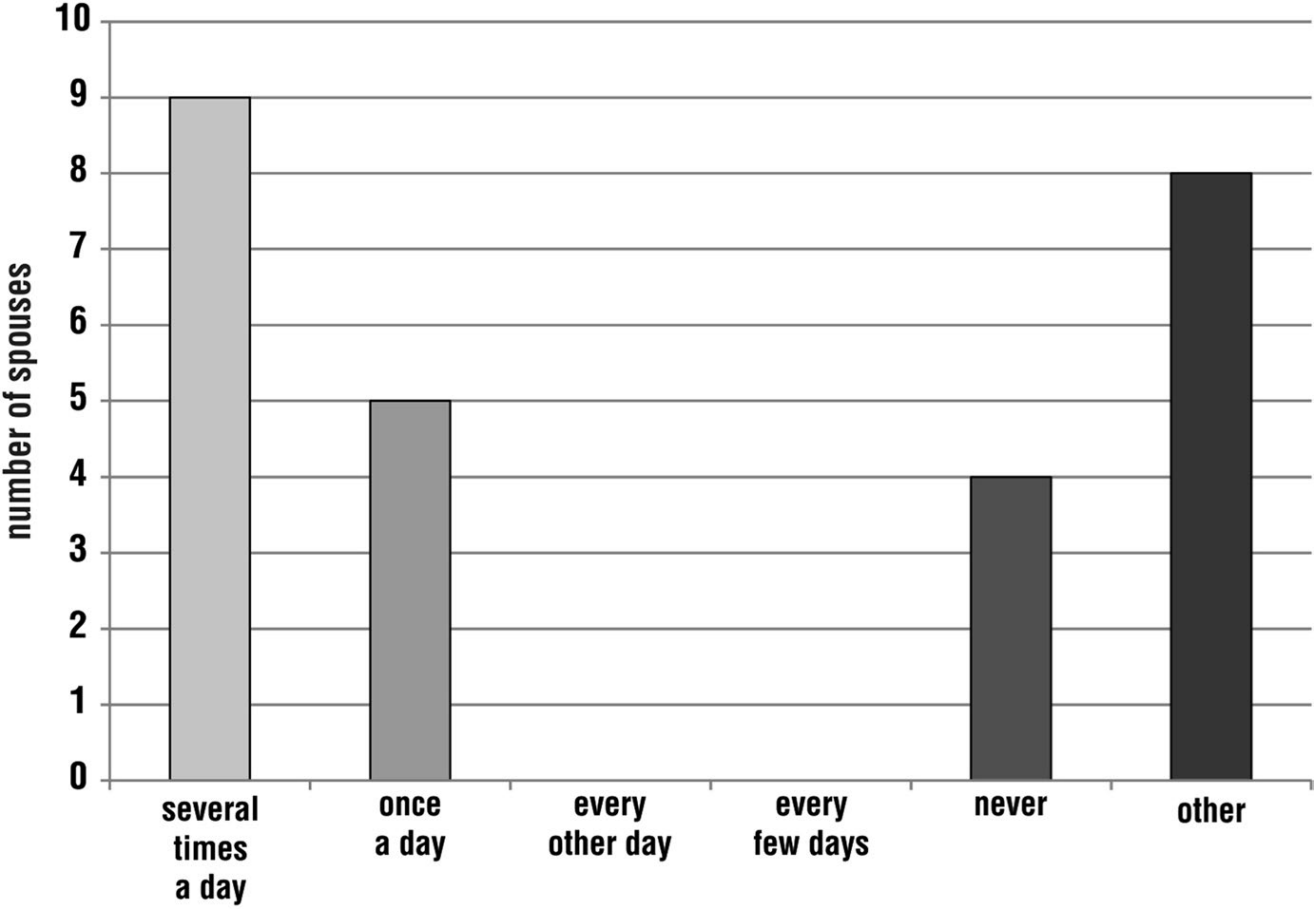
Distribution of respondents’ answers to the question, ‘Please indicate how often you prayed with your spouse while on pilgrimage’

During the pilgrimage, the spouses most often prayed together several times a day (nine responses; 30%). Some respondents chose the option ‘other’ (eight; 27%), adding, for example, that they did not go on the pilgrimage together, or that they prayed together only at Mass or only as part of a larger group of people. There was also a situation where a person could not estimate the frequency due to the lack of memory. Some respondents (four; 13%) admitted that they had never prayed with their spouse while walking the Camino. Another 17% (five) declared that prayer as a married couple took place once a day (Fig. 5).

**Fig. 6:**
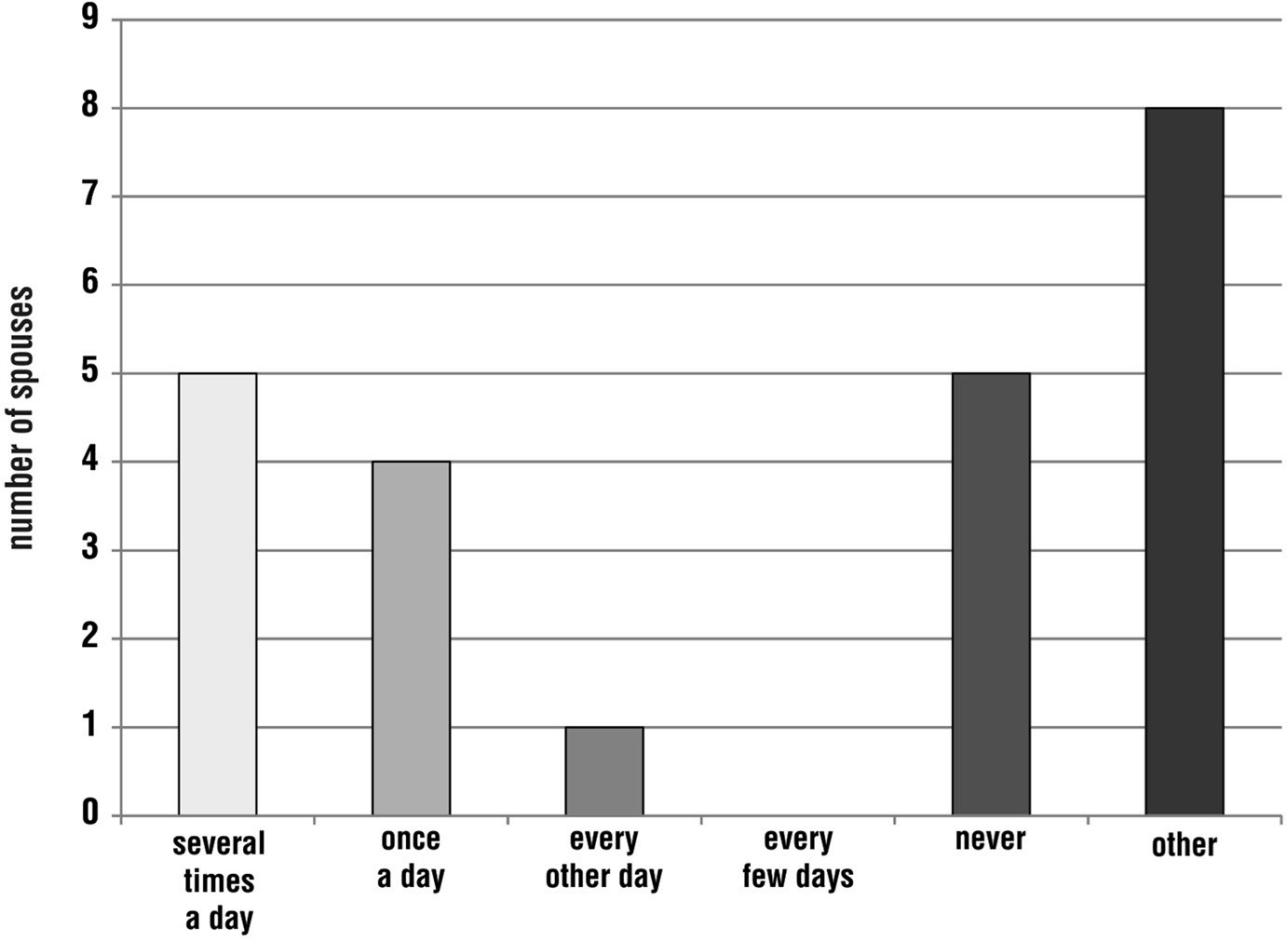
Distribution of respondents’ answers to the question, ‘Please indicate how often you prayed with your child(ren) while on pilgrimage’

Regarding family prayer, i.e. praying together with the child(ren), the respondents most often chose the option ‘other’ (eight; 27%), explaining, for example, that the child was too young to pray, they were still praying while singing, the joint prayer took place at the Eucharist or they could not specify the frequency. There were also pilgrimages without children, so the question did not apply to some respondents. The next largest group stated that praying with their child(ren) took place several times a day or never (five responses each; 17%). The option of praying once a day was indicated by four people (13%) and every other day by one person (3%) (Fig. 6).

### Changes in Behaviour and Relationships

The interview also included a question about new habits developed during the pilgrimage and applied to marital and family life after returning home. Twenty-six people responded, with six respondents unable to indicate or not noticing the emergence of new habits. In contrast, the remaining population identified such habits, among which the marital/familial and personal categories can be distinguished. The former includes habits such as spending more time together (seven responses), including more frequent conversations (also about their spouse’s needs and listening to them, as well as about religious topics), the practice of praying together (three), going out and walking together and making plans for the next pilgrimage (three), ‘revisiting the Camino’ by looking at a photo album (two) and increasing the independence of the children (one). In addition, the respondents spoke of the agreement, understanding, mutual patience and means of coping with life’s problems in relationships. On the other hand, among the changes in personal behaviour, the following can be singled out: consistency in the pursuit of one’s goals, calming down, speaking favourably of others and developing the habit of raising the intention with which one attends the Eucharist.

The respondents were also asked to describe how the pilgrimage had affected their marital or familial relationships. Twenty-seven responses were obtained. The spouses stated that going on the pilgrimage together brought them closer together and improved their relationship, which is ‘as warm as possible, full of love and understanding’ (P9); ‘we felt an even greater bond between us’ (P6). Communication improved through listening to each other, sharing experiences and taking time for each other. The pilgrimage made it possible to ‘sharpen’ the positive qualities they possessed, to appreciate strengths that the spouses did not always notice in everyday life. In addition, they reported becoming more sensitive and understanding of each other, which increased mutual understanding and improved their relationship. In the words of one respondent, ‘it was a certain step forward in the marital dialogue, an opportunity to be honest and show each other how much we mean to each other, and a motivation to work on each other’ (P15). The pilgrimage strengthened the family and the bond with the children by being together with each other and talking more often. Among the respondents, three people admitted that the pilgrimage had no impact on their marital or familial relationships. One response referred to distant family members not understanding the idea of pilgrimage.

### Difficulties in a Shared Pilgrimage

The respondents were also asked to describe what difficult moments in relationships (or on other levels) occurred during the pilgrimages with family members. Nineteen responses were obtained, with eight respondents declaring that there were no difficult moments that they could remember. On the other hand, when it came to such incidents at the relationship level, the respondents mentioned showing patience to their children, especially at times to motivate them to continue the pilgrimage when they felt tired; the child holding the parent responsible when the intended plan was not achieved; looking for accommodation of a minimum standard when making the pilgrimage with the child (however, in this case, the respondent added that often ‘for the children, these difficult conditions are the coolest adventure’ (P2); constantly spending time with their spouse despite their differences and previous relationship problems, and mastering negative emotions stemming from this; knowing that other pilgrims are watching their behaviour; and complaining and whining. Regarding difficult moments unrelated to relationships, the respondents mentioned tiredness, physical pain, worsened condition, finding accommodation, modest living conditions and finding time to be ‘alone with God’ due to the frequent contact with other pilgrims.

### The Impact of Pilgrimage on the Fusion of Marriage and Family Ties

When asked whether going on a pilgrimage together strengthened marital or familial relationships, 19 respondents (63%) marked the option ‘definitely yes’, eight (27%) chose the option ‘rather yes’, while three people (10%) did not give an answer. It is noteworthy that none of the respondents chose a negative answer.

The respondents were asked to describe how the pilgrimage had cemented the bonds of marriage and family. A total of 23 responses were obtained. The respondents acknowledged that going on a pilgrimage together strengthened bonds and mutual relationships, allowed them to get to know and understand their spouse/child better (‘it has united parents and children through time spent together, the journey together, prayer and the Eucharist’ [P16]), increased sensitivity to the other person, strengthened mutual trust, gave the feeling that the spouses can count on each other in any situation, taught gentleness and kindness, created opportunities to support and make time for each other, created a climate for talking and praying together (‘problems became lighter and more solvable’ [P6]) and made the spouses feel happy. Pilgrimages on the Camino de Santiago also had an impact on the spouse who did not make the journey, as by being away from the other, it allowed them to appreciate the tasks and responsibilities they performed on a daily basis. Only one person declared that the relationship remained at the previous level, suggesting that the pilgrimage did not play a role in the issue at hand.

The interview questionnaire also included a question about whether the pilgrimage along the Camino de Santiago had influenced the decision to have offspring. Twenty-four answers were obtained, of which 17 were negative and six explained that the issue no longer concerned them. This finding is likely related to the age of the survey group, as they were mostly people in long marriages who, consequently, had already had offspring. In contrast, one answer was in the affirmative: ‘In a way, yes, on our single Camino and on our family Camino we asked St James for our children; he listened’ (P2).

### Implanting the Spirit of Pilgrimage

The respondents were also asked how they intended to instil the spirit of pilgrimage in their own children. Twenty-eight people responded. Five of them explained that they did not need to carry out this task as their children were already on pilgrimage, either with them or separately as they grew up. Two respondents explained that their children used to go on pilgrimage, but for various reasons (e.g. starting a family of their own) they suspended the practice. Other respondents mentioned the following ways to instil the spirit of pilgrimage: talking about their own pilgrimage experience (Martínez et al., 2022), sharing their personal experiences and attitude towards life, inviting and motivating them to go on a pilgrimage together while respecting their decision, ‘pointing out to them the advantages and benefits of this form of spending time, through the joy and good humour that stays with us after the pilgrimage, praying for their children, so that they feel the need to look for real values’, spending time together and going on trips or parish pilgrimages to other places and showing the health (fitness) aspect of the pilgrimage to gradually show the spiritual and religious meaning. One person declared that he did not intend to instil the spirit of pilgrimage in his own children.

The respondents were asked to share how St James was an intercessor for them in marital or family matters. Out of 26 responses, eight respondents admitted that they either did not consider St James an intercessor in marital and family matters or had not thought of him that way until now. Above all, they emphasised the aspect of his intercession in matters of pilgrimage and the Camino itself, as well as invoking him in difficult life situations. To illustrate the different approaches of the other respondents, let the following statements serve as examples: ‘St James is definitely the recipient of more than one of my prayers for our family. I feel his protection. When we were worrying a lot about the health of our second son, it turned out that the date of delivery was set for 25 July—definitely not a coincidence’ (P2); ‘In every matter I turn to the help and intercession of St James, because he is my unfailing patron’ (P9); ‘St James is first and foremost the patron of my husband’s life. I am friends with my husband’s friend’ (P17); ‘He helps me to resolve difficult moments in my relationship with my husband’ (P12); ‘We belong to the Confraternity of St James and always ask him for discernment in choosing our path in life, so that we are always close to God and reject what distances us from him’ (P18); ‘To be faithful to each other and to make a pilgrimage together on this path that is marriage’ (P19); ‘He is the patron saint of our parish, and many families make the pilgrimage to improve marriage and family ties’ (P10); ‘Saint James is an intercessor in marriage and family matters … I would like Saint James to guard our family’ (P20); ‘Saint James reminds me that life is a journey, that marital and family life has clear and straight stretches, but even more twists and turns and moments where you have to trust in God and keep moving forward even though you don’t know how the road goes, you only know where you are going. So that we do not stop and do not deviate from the path marked out, even though we often do not have the strength to go on’ (P13).

**Fig. 7:**
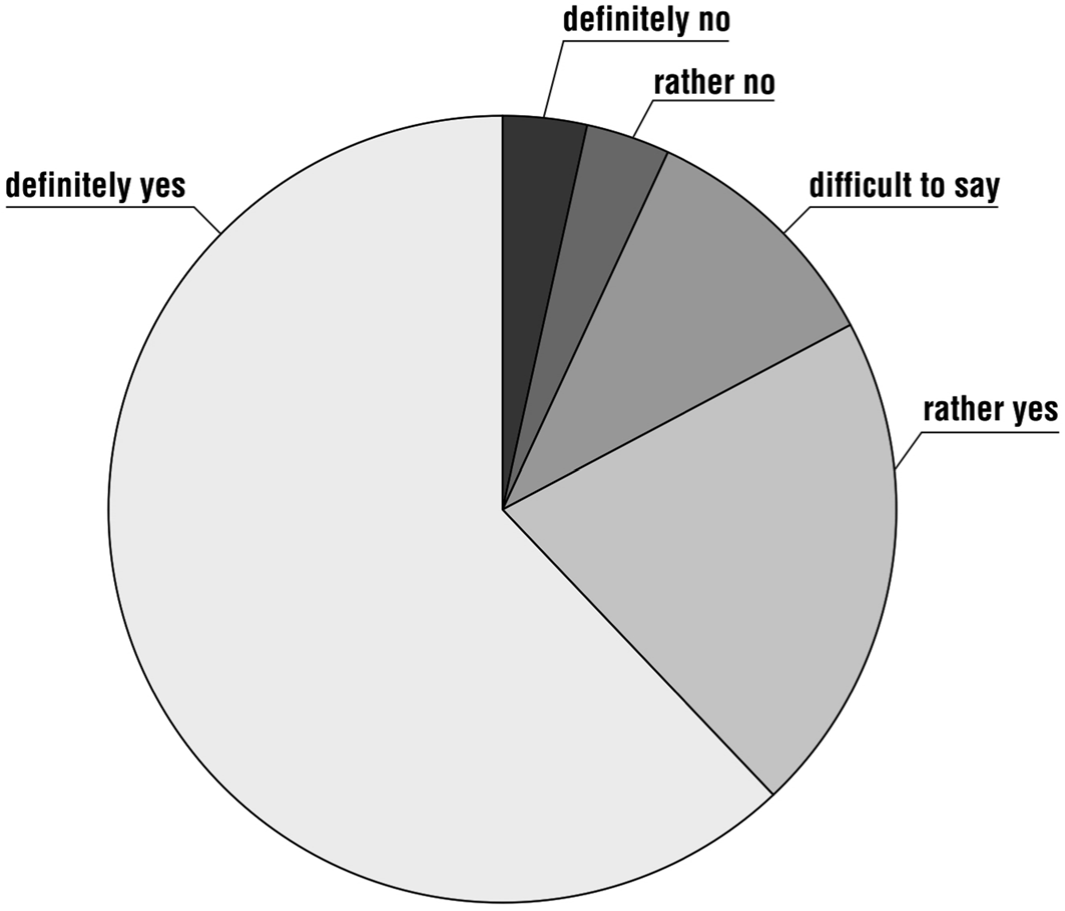
Distribution of respondents’ answers to the question, ‘Please indicate if you plan to go on pilgrimage again with your spouse/child(ren)’

When asked whether the respondents plan to go on a pilgrimage again with their spouse or child(ren), 18 people (60%) answered ‘definitely yes’, six (20%) answered ‘rather yes’, three (10%) chose ‘it is difficult to say’ and one person each (3%) stated ‘rather no’, ‘definitely no’ and did not answer the question (Fig. 7).

## Study Limitations

It is important to emphasise that studies of the impact of shared pilgrimage on the Camino de Santiago on marital or familial relationships are not representative. Despite this limitation, thanks to the research, observations and in-depth interviews conducted since 2006 in the Camino de Santiago pilgrimage space in Europe—over 8,000 km of the Way of St James in Spain, Portugal, France, Poland, Czechia, Germany, Austria, Switzerland, Slovakia, Estonia, Latvia and Lithuania—it is possible to transfer these results to other sections of the Camino de Santiago and to anchor them in the literature. It would certainly be interesting to compare these results with studies of marital/familial relationships on other routes, such as hikes in national parks (Hyelin et al., 2015).

## Conclusions and Discussion

This study, in the form of a survey, contributes to the literature on the Way of St James insight into how pilgrimage along the Camino de Santiago as families or married couples affects their marital or familial relationships and shapes the attitudes and behaviour of pilgrims. The analysis clearly shows the positive aspect of going on a pilgrimage together. The interviewed spouses admitted that the pilgrimage helped to strengthen their relationship. This was influenced by the time spent together and the many conversations that could not have been held in the hustle and bustle of everyday life. The pilgrimage became a space for the spouses to meet and gift each other with their presence. This translated into long, quality conversations and valid topics discussed. The constant contact allowed the spouses to get to know each other better and to become emotionally closer. Shared pilgrimage prevented relational escapism. The spouses could not fail to bring up difficult topics that they ran away from on a daily basis. They had to discuss them and work out a solution together. Sometimes this was also helped by talking to other spouses they met, which allowed them to see their relationship from a different perspective. It could be said that going on a pilgrimage together took on a therapeutic dimension, helping to make personal and relational insights and resulting in changed behaviour and improved relationships. The time spent together and the conversations held translated into new interactions that the spouses implemented after returning to everyday life. This further led to improved communication, mutual understanding, patience and problem-solving. In addition, hiking together made it possible to discover and appreciate each other’s diversity and difference and learning and understanding each other’s needs, which contributes to building a marital community.

The results indicate that joint pilgrimage along the Way of St James by married couples and parents with children strengthens marital and familial relationships. A joint pilgrimage along the most beautiful road in the world, as the Camino de Santiago is called, brings spouses closer together, improves relationships, enhances communication and allows them to appreciate their spouse’s strengths, which the ‘other half’ does not always notice in everyday life. On the other hand, journeying with other couples also helps the spouses to appreciate the value of their marriage and allows them to realise that every marriage faces various difficulties and is not perfect and flawless in every dimension, and that mistakes or even downfalls must be learnt from and worked through so that they are not repeated. Pilgrimage as a family allows parents to get to know their children better and to solidify their relationship with them. During the Camino de Santiago, spouses give each other support—both physical and psychospiritual. Both of these forms of support are of great importance for spouses and their children. Surveys show that the conversations they have together on the Camino are extremely valuable, ‘for which there is finally time, far away from daily obligations’—conversations that are balanced, thoughtful and, more than in everyday life, free of extreme negative emotions.

In addition to the conversations, gestures of kindness were also an element that strengthened bonds. These most often manifested in concern for the psychophysical condition of one’s spouse, motivating them to overcome the journey in moments of fatigue and resignation, preparing a meal, carrying equipment and putting on bandages, which became a manifestation of tenderness and love. It was also important to pray together as they made their way along the road. It is noteworthy that the maintenance of conjugal prayer was perpetuated and took place even after the pilgrimage was over. Thus, making the pilgrimage together made the spouses more sensitive to each other, increased mutual trust and the feeling that they could count on each other. They became more understanding, patient, gentle and kind to each other and realised how much they meant to each other.

In addition to the impact of pilgrimage on marital relationships, the study also included the impact on familial relationships with children. Among those surveyed, 40% stated that they had travelled at least some part of the journey with their offspring. The most common were teenagers between the ages of 13 and 18, followed by children aged 6–12, then children aged 0–5 and adult children. The respondents found that going on a family pilgrimage allowed them to get to know their children better and reinforce their positive relationships with them. Going on a pilgrimage together contributed to the discovery of children’s hitherto unnoticed qualities and learning about their interests and views. Sometimes parents also noticed the need to change their behaviour towards their offspring, to accept their individuality and to develop an equal parenting style with their spouse. Above all, the interviewed parents valued spending time with their children, especially their teenagers, as they did not have as many opportunities to talk and be with each other on a daily basis. Shared contact with parents protects adolescents from alienation, loneliness, anomie and a kind of family ‘homelessness’ (Pilarz et al., 2019).

Reflecting on the impact of the Camino de Santiago pilgrimage on marital-familial relationships and on the formation of attitudes and behaviours, it should be noted that the pilgrimage itself is not the only predictor of a positive evaluation. The study included spouses who, at the time of the pilgrimage, had a long (over 20 years) or rather long (11–20 years) marriage. Thus, these are relationships with experience of living together, building relationships and overcoming difficulties or crises. The individuals had already developed mechanisms to function together, form bonds and take care of the emotional sphere of the relationship. All this becomes a resource that influences the condition of the marriage. Thus, pilgrimage on the Camino de Santiago cannot be treated as an unproven means for improving marital or familial relations.

It can be assumed that there are other conditions for pilgrimage to have a positive effect. Apart from the love of spending time on such an activity, religiousness is a favourable circumstance. This thesis is confirmed by the homogeneity of the population, since 100% of the respondents were Catholics. Their attitude to faith was also similar, with the majority of respondents describing themselves as believers (53%) or firm believers (43%), as was the type of relationship they were in (97% were in a sacramental relationship). These similarities may be linked to and reflected in the motives for the decision to make the pilgrimage, as religious and spiritual motives predominated. On the other hand, the positive assessment of family pilgrimage cannot be influenced by the profession of the respondents, as they present a whole cross-section of different professions, nor by their material situation or level of education, as diversity was noted there as well.

In conclusion, going on the pilgrimage of the Camino de Santiago as a family or married couple had a positive impact on intra-family relationships. According to the interviewees, going on the pilgrimage together deepened marital relationships, strengthened bonds and trust, improved communication and knowing each other better and helped show care and tenderness. Also, it had a positive impact on contact with the children through time spent together and conversations, which allowed them to get to know each other better, show mutual interest and see each other’s needs.

The pilgrimage space of the Camino de Santiago, as with other long-distance pilgrimage routes, provides full opportunities for personal development and for the development of bonds between spouses, between parents and children, between siblings and between friends (Echarri, 2021). The space of pilgrimage walking along the Way of St James has been used for more than 20 years in many countries around the world for educational and rehabilitation walking projects aimed at getting young offenders out of a criminogenic environment, teaching them self-reliance and making them realise that their future depends mainly on them. Such projects are implemented in France, Germany, Poland, Switzerland, Italy, Hungary and South Korea (Mróz & Matuszczak, 2019).

Our experience as pilgrims, as well as our research, confirms that pilgrimage—especially this walking pilgrimage–is hugely beneficial for mental health and promotes emotional, spiritual, intellectual, social and physical well-being (Eade, 2000; Faria et al., 2022; Jørgensen et al., 2020; Klimiuk & Moriarty, 2021; Mikaelsson, 2012). This well-being, in turn, improves the pilgrim’s relationship with loved ones—spouse, children or friends—during the pilgrimage, and, perhaps most importantly, when the pilgrim returns home to everyday responsibilities with recharged spiritual and psychophysical batteries (Vidal-González & Sánchez, 2019).

Pilgrimage and walking the Camino de Santiago offer great opportunities for many pastoral, evangelical and social initiatives as well, perhaps especially for couples and families.
